# Supplementary material for: Nanocomposite of Fullerenes and Natural Rubbers: MARTINI Force Field Molecular Dynamics Simulations
Source: Polymers (Basel). 2021 Nov 22;13(22):4044. doi: 10.3390/polym13224044 (PMC8626026; doi:10.3390/polym13224044)
Supplement: Supplementary file 1 [file polymers-13-04044-s001.zip › polymers-1438885-supplementary.pdf]

polymers MDPI

Supporting Information:

Nanocomposite of Fullerenes and Natural Rubbers: MARTINI Force Field Molecular Dynamics Simulations

Jiramate Kitjanon [1,2], Wasinee Khuntawee [1,2,3], Saree Phongphanphanee [2,3,4], Thana Sutthibutpong [2,3,5], Nattaporn Chattham [1], Mikko Karttunen [6,7,8] and Jirasak Wong-ekkabut [1,2,3,*]

[1]Department of Physics, Faculty of Science, Kasetsart University, Bangkok 10900, Thailand

[2]Computational Biomodelling Laboratory for Agricultural Science and Technology (CBLAST), Faculty of Science, Kasetsart University, Bangkok 10900, Thailand

[3] Thailand Center of Excellence in Physics (ThEP Center), Commission on Higher Education, Bangkok 10400, Thailand

[4] Department of Material Science, Faculty of Science, Kasetsart University, Bangkok, 10900, Thailand

[5] Department of Physics, Faculty of Science, King Mongkut's University of Technology Thonburi (KMUTT), Bangkok 10140, Thailand

[6] Department of Chemistry, The University of Western Ontario, 1151 Richmond Street, London, ON, N6A 3K7, Canada

[7] Department of Physics and Astronomy, The University of Western Ontario, 1151 Richmond Street, London, ON, N6A 3K7, Canada

[8] The Center for Advanced Materials and Biomaterials Research, The University of Western Ontario, 1151 Richmond Street, London, ON, N6A 3K7, Canada

*Correspondence: jirasak.w@ku.ac.th (J.W.)

Table S1: Details of the CG force field for *cis*-PI.

Rubber chain structure

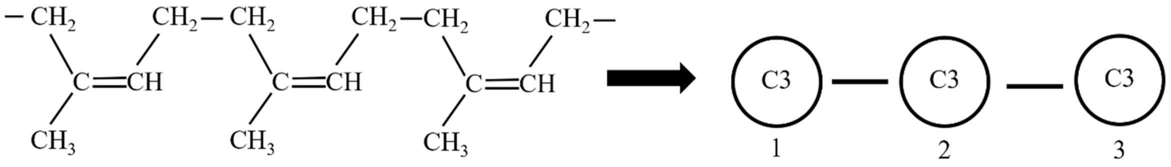

**Bonded interaction**		k_b (kJmol^{-1}nm^{-2})	b_0 (nm)
1-2		5000	0.46
2-3		5000	0.46
**Angle bending**		k_θ (kJmol^{-1})	θ_0 (degree)
1-2-3		37	110
**Non-bonded interaction: Lennard-Jones potential**			
		C6	C12
C3	C3	0.15091	0.0016267

Table S2: Number of molecules and simulation time.

Fullerene Concentration (phr)	Number of Fullerene (molecule)	Number of *cis*-PI (chain)	Simulation time (μs)
0	0	500	21
4	53	500	21
8	107	500	21
16	213	500	21
32	427	500	21

Table S3: Solvation free energy of an added fullerene in water, *cis*-PI in melt and composite at different C_{60} concentrations.

Solvate	Free energy (kJ/mol)	Reference
Water	-99.53 ± 0.03	-92.6 [1]
Melt	-215.08 ± 0.14	
4phr	-214.37 ± 0.10	
8phr	-213.95 ± 0.29	
16phr	-212.40 ± 0.13	
32phr	-210.43 ± 0.33	

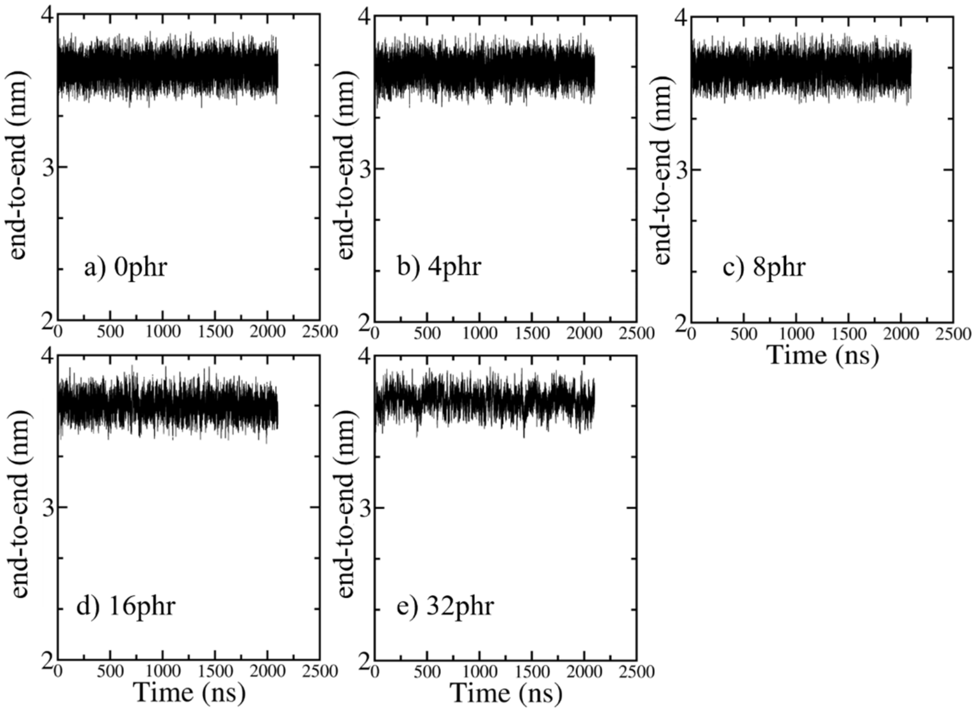

Figure S1: Time evolution of end-to-end distance (R_0) with varies C_{60} concentration from 0 to 32 phr.

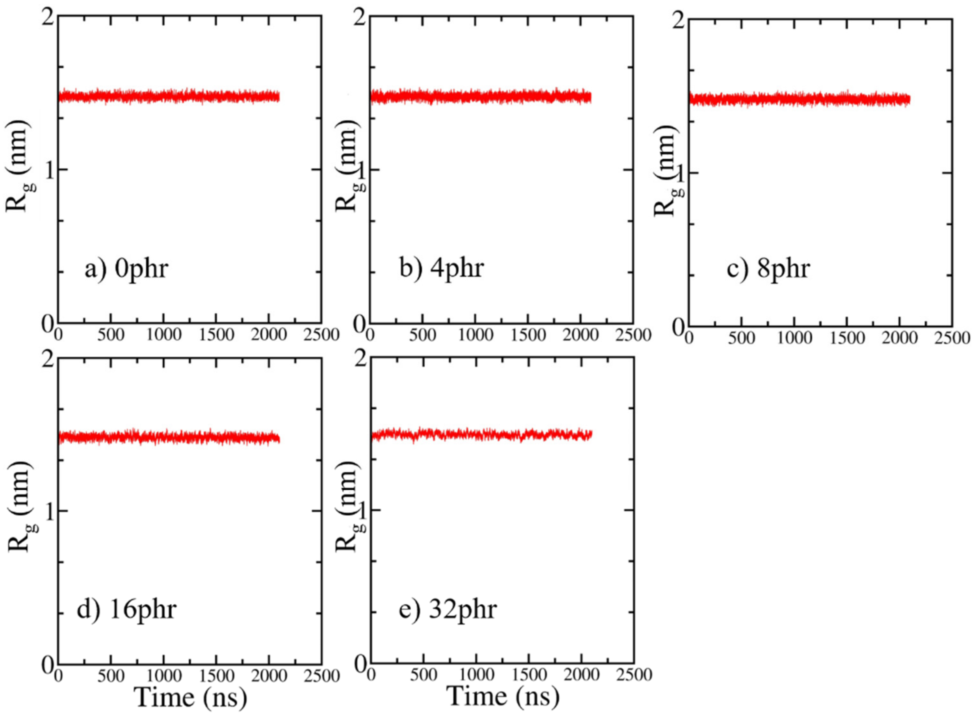

Figure S2: Time evolution of radius of gyration (R_g) with varies C_{60} concentration from 0 to 32 phr.

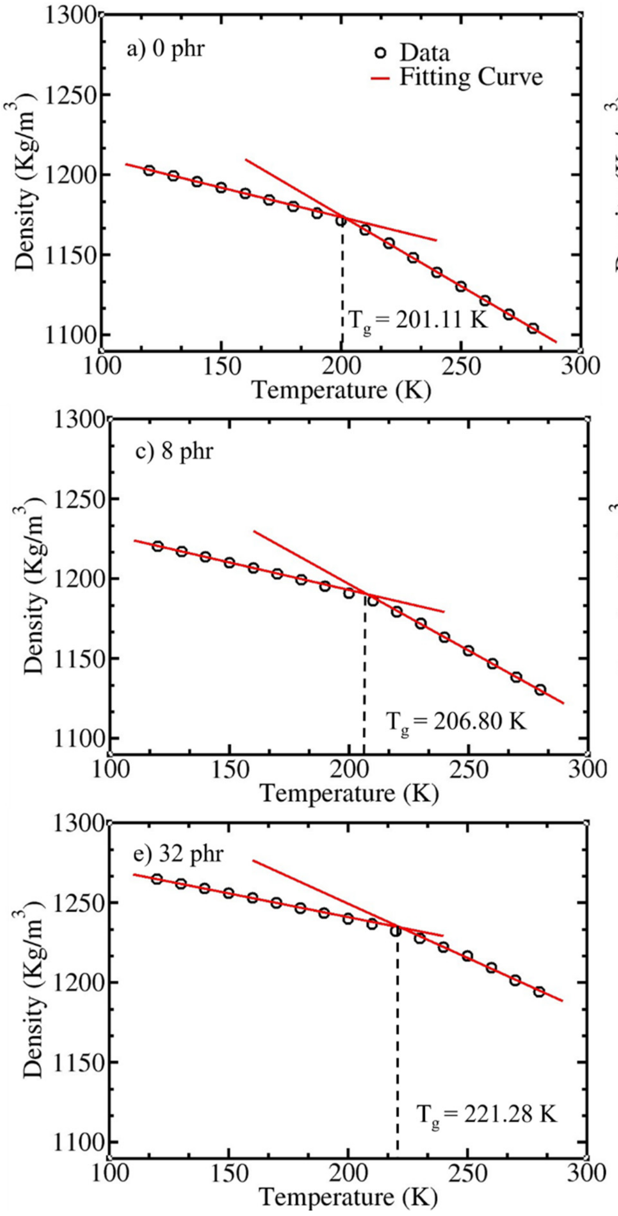

Figure S3: Density versus temperature of *cis*-PI-C_{60} composites.

Reference

1. Nisoh, N.; Karttunen, M.; Monticelli, L.; Wong-Ekkabut, J. Lipid monolayer disruption caused by aggregated carbon nanoparticles. *RSC Adv*. **2015**, *5*, 11676-11685.
